# A novel technique for implementing the finite element method in a shallow water equation

**DOI:** 10.1016/j.mex.2023.102425

**Published:** 2023-10-09

**Authors:** Putu Veri Swastika, Muhammad Fakhruddin, Sofihara Al Hazmy, Siti Fatimah, Amaury de Souza

**Affiliations:** a Department of Mathematics, Faculty of Mathematics and Natural Sciences, Universitas Udayana, Jl. Raya Kampus UNUD, Bukit Jimbaran, Kuta Selatan, Badung 803611, Bali, Indonesia; b Mathematics Department, School of Computer Science, Bina Nusantara University, Jakarta 11480, Indonesia; c Department of Mathematics, Faculty of Defense Science and Technology, Universitas Pertahanan Republik Indonesia, Kawasan IPSC Sentul, Sukahati, Bogor, Jawa Barat 16810, Indonesia; d Mathematics Study Program, Faculty of Mathematics and Science Education, Universitas Pendidikan Indonesia, Dr. Setiabudi street, Bandung, 40154, Indonesia; e Federal University of Mato Grosso Do Sul, CP 549, 79070-900, Campo Grande, MS, Brazi

**Keywords:** Finite element method, Non-conformal basis, Shallow water equation, *The*$P_{1}^{NC}\;-P_{1}$*Finite Element Method*

## Abstract

We presented a novel approach to investigate the two-dimensional shallow water equation in its primitive form. Its employs the $P_{1}^{NC}-P_{1}$ element pair to simulate various cases: standing waves, dam-break planar, and wave absorbing with embedded radiation boundary conditions. Unlike the conventional method, we approximate the free surface variable using a conformal basis $P_{1}$ whereas the velocity potential is approximated using a non-conformal basis, $P_{1}^{NC}$. Thus, for each case, the weak form needs to be reformulated as well as the discrete form. The resulting scheme is a first-order ordinary differential system and solved by Crank Nicholson. The mass matrix in the momentum equation contains the multiplication between the two bases, which computed by the mass lumping. So, our method is explicit, flexible and easy to implement. Validation using standing waves demonstrated first-order accuracy, free from numerical damping and convergent to the analytical solution. Dam-break simulation result shown an agreement with ANUGA software. Our scheme's flexibility is demonstrated when it can mimic wave absorbing simulation employing embedded radiation boundary conditions. The reflection at the boundary seems small enough, thus can be neglected. All these findings have shown the robustness and capability of our scheme to predict accurate results for various shallow water flow problems.

• A novel technique for solving 2D SWE in primitive form
• It is explicit, flexible, easy to implement, accurate, and robust
• Our approach is suitable for coastal/oceanographic simulations

## Graphical abstract

**Figure:**
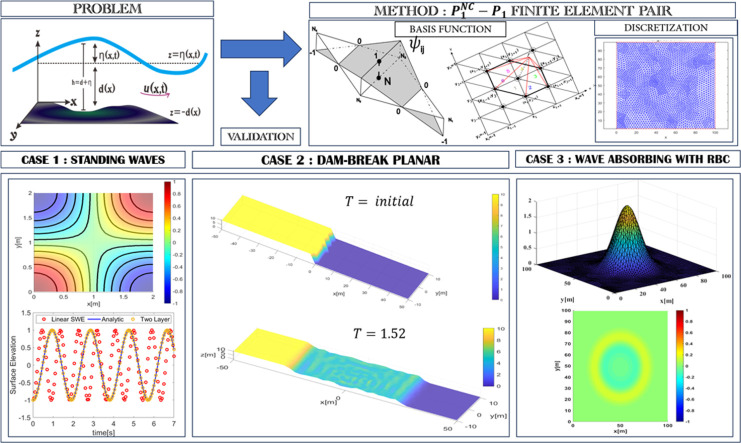

Specifications table

**Table:** 

Subject area:	Mathematics and Statistics
More specific subject area:	*Computational Fluid*
Name of your method:	*The*$P_{1}^{NC}\;-P_{1}$*Finite Element Method*
Name and reference of original method:	*B. -L Hua and F. Thomasset, A noise-free finite element scheme for the two-layer shallow water equations, Tellus A,36 A(2)157–165, 1984.*
Resource availability:	*N.A.*

## Introduction

The shallow water wave equation (SWE) is a partial differential equation that enables us to gain a deeper understanding of physical phenomena in shallow environments. Assuming the horizontal length scale is greater than the depth scale, the SWE widely use to describe various real-world phenomena, for example: standing waves [[1], [2], [3], [4]], wave refraction [5], dam break [6], internal wave generation in the strait [[7], [8], [9], [10]], tsunami propagation in near-shore areas [11], and etc.

The solution of SWE can be determined using either the analytical method or the numerical method. However, the analytical solutions to SWE are only available for specific conditions with ideal assumptions. Therefore, it is crucial to establish reliable, efficient, and practical numerical methods for real-world applications. Extensive study on numerical models for SWE has been conducted, which can be broadly classified into three categories, i.e., the finite element technique [12], the finite difference method [13], and the finite volume approximation [14].

Physical occurrences in shallow environments frequently involve irregular geometric shapes, which necessitates the use of numerical methods capable of handling complicated geometric shapes. The finite element approach is one method that offers advantages when dealing with complicated geometric shapes. In recent decades, there have been several attempts to develop finite element numerical methods. However, various challenges exist, including the problem of spurious computational modes. This problem can arise for certain choices of grids and bases due to the coupling between the momentum and continuity equations [15]. To prevent these modes, two ways have been proposed: (a) modifying the SWE to remove the troublesome terms or (b) determining the correct discretization (using Finite Element pairs). In the first option there are several studies that modify the SWE equation, including: vorticity and divergence formulations [[16], [17]] and a wave-equation formulation [[18], [19]]. The wave-equation formulation was first proposed by Lynch and Gray, [19] to correctly handle the wave propagation issues. This wave equation formulation, on the other hand, appears to have several issues, such as advective instabilities and mass conservation [[20], [21]]. This problem with the first option regarding the formulation of the wave equation, led to further research on the second option, determining the correct discretization using finite element method to solve primitive equations without using modified formulations or stabilization. However, the approach with the conventional finite element method is also not easy given the same approximation location for surface and velocity variables [15]. For this reason, an approach using the finite element pair method, where the different location approximation for both surface and velocity variable are suggested.

Several emerging finite element pairs are gaining popularity due to their benefit of not experiencing spurious pressure modes. One particular is $P_{1}^{NC}-P_{1}$ finite element pair which use linear non-conforming approximation ($P_{1}^{NC})$ for velocity and a linear conforming approximation ($P_{1}^{NC})\;$for elevation. Crouzeix and Raviart [22] proposed non-conforming finite elements to solve Stokes equations which proved to be well suited to represent transport processes. This element is referred to as non-conforming because it is continuous only across triangle boundaries at midpoint nodes and discontinuous everywhere else around a triangle boundary [[23], [24]]. Furthermore, Hua and Thomasset [25] studied the combination of linear conforming and non-conforming finite elements to solve the shallow water equations. They found that this finite element pairs is computationally efficient and properly models the dispersion of the inertia-gravity waves [26]. The advantage of using these two basis functions simultaneously is that they are staggered in space [[24], [27]], computationally efficient due to orthogonal matrices [26] and the commonly used approach has proven to be unconditionally stable [27].

In this paper, we propose a novel technique to solve SWE using finite element method (FEM) based on our previous research [28]. In our prior work, we proposed a novel one-dimensional basis function, the discontinuous (non-conformal) $P_{1}^{NC}$. Our study was inspired by Hua and Tommaset [25], the $P_{1}^{NC}-P_{1}$ finite element pair for solving the two-dimensional SWE equation. We found that a discontinuous one-dimensional basis function $P_{1}^{NC}$ does not yet exist, despite the fact that its two-dimensional basis is well-established. The nature of the $P_{1}$ shape function is to have a value of 0 for all neighboring nodes but a value of 1 at one node. Meanwhile, the non-conforming $P_{1}^{NC}$ shape functions have a value of 1 at one edge, linearly change to −1 at the opposite node, and have a value of zero at the midpoint of the other two edges [[25], [27]]. In this present work, we extend our previous approach to solve 2D SWE. Here we solve the two-dimensional SWE equation in its primitive form. As suggested by Le Roux et al. [15], the use of finite element pairs together with primitive equations may give some benefit. In contrast to the common approaches given by several authors [[25], [26], [27]], here, we approximate the free surface variable using a conformal basis $P_{1}$, while the velocity potential is approximated using a non-conforming basis $P_{1}^{NC}$. Thus, the weak formulation needs to be reformulated again, as well as the discrete scheme. Furthermore, we show the capability of our proposed scheme by simulating various problems, including: the standing waves, and dam-break planar 2D. The accuracy of our scheme is validated with the analytical solution of classical standing waves. Next, the dam-break simulation are performed and compared with ANUGA software [29]. Finally, wave absorbing simulation are presented and discussed to investigate the flexibility of our modified numerical scheme with radiation boundary condition embedded.

The organization of this paper are as follows. The governing equations, the weak form and the discrete form are discussed in Section 2. Here we reformulate the weak formulation according to the primitive form of SWE, together with the formulation of hard-wall boundary condition. The discrete form is constructed where the free surface is approximated by a conformal basis $P_{1}$, but the velocity potential is approximated by a non-conformal basis $P_{1}^{NC}$. In Section 3, we apply our numerical scheme to study various 2D cases, including standing waves, dam-break planar, and the implementation of absorbing radiation boundary conditions (RBC). We modified our proposed weak form due to the use of RBC. We focus on the performance of our numerical scheme. In Section 4, we investigate the influence of absorbing boundary conditions on the weak formulation and discrete form of our proposed method as well as its numerical implementation. Finally, conclusions and remarks will be given in the last section.

## Method details

This section describes the $P_{1}^{NC}-P_{1}$ finite element method, including the mathematical model and finite element discretization.

### Two-dimensional SWE

Let's consider a three-dimensional spatial coordinate system with vertical coordinate z, horizontal coordinates x=(x,y), and time coordinate t. Here we assume a layer of ideal fluid bounded below by an impermeable topography z=d(x)and bound above by a free surface z=η(x,t). Total depth is denoted by h(x,t)=d(x)+η(x,t) whereas u(x,t) denotes the horizontal velocity of the fluid particles. Here we assume that fluid flow is hydrostatic, so the vertical velocity of the fluids is negligible. Assuming the horizontal length scale is greater than the depth scale, the fluid motion in the shallow area is governed by a pair of mass conservation and momentum balances, or Shallow Water Equations (SWE) as follows.

(1) $$\frac{\partial h}{\partial t}+\nabla .\left(hu\right)=0,$$

(2) $$\frac{\partial u}{\partial t}+u.\nabla u+g\nabla h=-C_{f}\frac{u\left|u\right|}{h}.$$

where $g=9.8m/s^{2}$ is the gravitational acceleration, $C_{f}$ is coefficient of friction, and $\nabla =(\partial_{x},\partial_{y})$ is the differential operator. If we consider a linearized SWE with a non-moving bottom and without friction, then we have

(3) $$\frac{\partial \eta }{\partial t}+\nabla .\left(hu\right)=0,$$

(4) $$\frac{\partial u}{\partial t}+g\nabla \eta =0.$$

If we assume that the flow is irrotational, then curl $u\;\equiv \;\nabla_{3}\;\times u\;=\;0$, with $\nabla_{3}=\left(\nabla ,\partial_{z}\right)$. So there exists a function (scalar) velocity potential Φ(x,z,t) such that $u\;=\;\nabla_{3}\Phi \;=\;\left(\nabla \Phi ,\;\partial_{z}\Phi \right).$ If we consider that Φ doesn't depend on z, then Φ(x,z,t)≈Φ(x,z=η(x,t),t)=ϕ(x,t). So, we can rewrite [[22], [27]] in primitive form

(5) $$\frac{\partial \eta }{\partial t}+\nabla .\left(h\nabla \phi \right)=0,$$

(6) $$\frac{\partial \phi }{\partial t}+g\eta =0.$$

In contrast with the common approach, here we will solve the 2D linear SWE [[1], [16]] by using $P_{1}^{NC}-P_{1}\;$finite element pair.

### $P_{1}^{NC}-P_{1}$ finite element method

In order to derive the scheme in the finite element sense, we start by formulating the variational problem of the governing equations [[1], [16]]. Let $\Omega_{p}=\left[x_{min},x_{max}\right]\times \;\left[y_{min},y_{max}\right]$ be the spatial domain with boundary $\partial \Omega_{p}.$ Suppose the test functions of the equations [16] and [1] are V(x), W(x) in suitable test spaces E and P respectively. If we multiply [[1], [16]] by V(x), W(x) respectively, and integrate along the computational domain $\Omega_{p}$, we obtain the weak form

(7) $$\int_{\Omega_{p}}\partial_{t}\eta Vd\Omega_{p}=-\int_{\Omega_{p}}\nabla .\left(d_{0}\nabla \phi \right)\;Vd\Omega_{p}$$

(8) $$\int_{\Omega_{p}}\partial_{t}\phi Wd\Omega_{p}=-g\int_{\Omega_{p}}\eta Wd\Omega_{p}$$

for flat $d\left(x\right)=d_{0}$. If we extend the R.H.S. of [30] using Gauss's theorem, we obtain the expression containing the velocity potential gradient, representing the contribution of the employed boundary conditions on $\partial \Omega_{p}$. To remove the velocity potential gradient, we employed a hard-wall boundary and obtained the weak form: *determine* η∈E *and* ϕ∈P

(9) $$\int_{\Omega_{p}}\left(\partial_{t}\eta V-d_{0}\nabla \phi \nabla V\right)d\Omega_{p}=0,\;\forall V\in E,$$

(10) $$\int_{\Omega_{p}}\left(\partial_{t}\phi +g\eta \right)Wd\Omega_{p}=0,\;\forall W\in P.$$

**Fig. 1:**
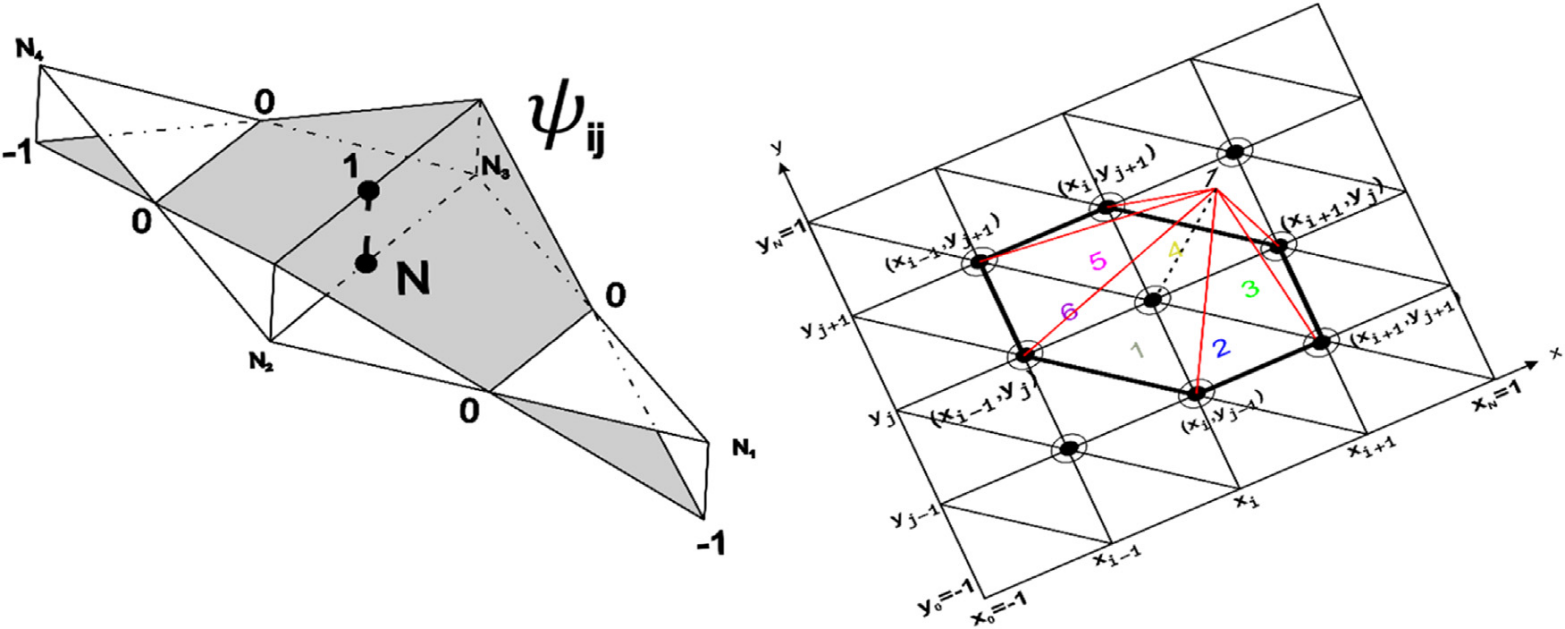
The basis function used in $P_{1}^{NC}-P_{1}$ finite element method. (left) $P_{1}^{NC}$ basis function redrawn from Daniel Y. Le Roux. [30]., (right) $P_{1}$ basis function.

In the derivation of the finite element scheme, we use a Galerkin procedure to approximate the weak form [[11], [26]] by writing η as a linear combination of conformal linear basis $P_{1}$ or standard linear basis ${\left\{T(x)\right\}}_{0}^{N_{V}}\;$and by writing ϕ as a linear combination of non-conformal linear basis $P_{1}^{NC}\;$or simply, discontinuous linear basis ${\left\{\psi (x)\right\}}_{0}^{N_{S}}$ such that

(11) $$\eta \left(x,t\right)≅\sum_{k=1}^{N_{V}}\eta_{k}\left(t\right)T_{k}\left(x\right),$$

(12) $$\phi \left(x,t\right)≅\sum_{k=1}^{N_{S}}\phi_{k}\left(t\right)\psi_{k}\left(x\right),$$

where $\eta_{k},\;\psi_{k}$ denote nodal values and $N_{S},\;N_{V}$ denote the number of segments and vertices of the triangles respectively. The discontinuous linear basis ${\left\{\psi (x)\right\}}_{0}^{N_{S}}$ and standard linear basis ${\left\{T(x)\right\}}_{0}^{N_{V}}$ is illustrated as in Fig. 1.

Substituting [[18], [25]] into the weak formulation [[11], [26]], we have the discrete form given by the following expression

(13) $$\partial_{t}\sum_{k=1}^{N_{V}}\eta_{k}\left(t\right)\left[\int_{\Omega_{p}}T_{k}\left(x\right)T_{j}\left(x\right)\right]d\Omega_{p}=d_{0}\sum_{k=1}^{\max\left(N_{V},N_{S}\right)}\phi_{k}\left(t\right)\left[\int_{\Omega_{p}}\nabla \psi_{k}\left(x\right)\nabla T_{j}\left(x\right)\right]\;d\Omega_{p},$$

(14) $$\partial_{t}\sum_{k=1}^{N_{S}}\phi_{k}\left(t\right)\left[\int_{\Omega_{p}}\psi_{k}\left(x\right)\psi_{j}\left(x\right)\right]d\Omega_{p}=-g\sum_{k=1}^{\max\left(N_{V},N_{S}\right)}\eta_{k}\left(t\right)\left[\int_{\Omega_{p}}T_{k}\left(x\right)\psi_{j}\left(x\right)\right]d\Omega_{p}.$$

The discrete forms given by [[8], [20]] can be written in the

(15) $$\left(\begin{array}{cc} M & 0\\ 0 & M_{\mathrm{nc}} \end{array}\right)\partial_{t}Y\left(t\right)=\left(\begin{array}{cc} 0 & S\\ -g\bar{M} & 0 \end{array}\right)Y\left(t\right)$$

where the element of mass matrices $M,\;M_{\mathrm{nc}}$ and stiffness S formulated as

$$Y\left(t\right)={\left[\eta_{1},\eta_{2},\ldots ,\eta_{N_{V}},\;\;\phi_{1},\phi_{2},\ldots ,\phi_{N_{S}}\;\right]}^{T},\;M_{\mathrm{nc}}=\left[m_{kj}^{*}\right],m_{kj}^{*}=\int_{\Omega_{p}}\psi_{k}\left(x\right)\psi_{j}\left(x\right)d\Omega_{p},$$

$$M=\left[m_{kj}\right],\;m_{kj}=\int_{\Omega_{p}}T_{k}\left(x\right)T_{j}\left(x\right)d\Omega_{p},\;\bar{M}=\left[m_{kj}\right],\;m_{kj}=\int_{\Omega_{p}}T_{k}\left(x\right)\psi_{j}\left(x\right)d\Omega_{p},$$

$$S=\left[s_{kj}\right],\;s_{kj}=\int_{\Omega_{p}}d\left(x\right)\nabla \psi_{k}\left(x\right)\nabla T_{j}\left(x\right)d\Omega_{p}.$$

where the integral can be evaluated numerically using the trapezoidal method. The term d(x) in S appears when we consider the varying topography. Furthermore, the R.H.S of [20] contains the multiplication between two different bases where both the base functions and test functions sum to 1.0 everywhere, and all terms in the mass matrix of an element will sum to the surface area of that element [31]. Then, we can use the mass lumping technique to obtain $\bar{M}=M_{nc}$ see the detailed description in [[26], [27], [31]]. Furthermore, the resulting scheme [12] is an ordinary differential system that can be solved by any time integration method. The procedure of $P_{1}^{NC}-P_{1}\;$finite element pair approach for solving 2D- SWE is described as follows:

1. Derive the variational problem of 2D SWE in primitive form to reformulated the weak form
2. Employs the Galerkin procedure to get the discrete form. Here, we approximate the free surface η as a linear combination of conformal linear basis $P_{1}$ and the velocity potential ϕ as a linear combination of non-conformal linear basis $P_{1}^{NC}.$
3. Compute the mass and stiffness matrix using any numerical integration method.
4. Solve the discrete form which is ODE system using any time integration method. Here we use Crank-Nicholson time integration scheme.
5. The $P_{1}^{NC}-P_{1}\;$finite element pair is evaluated by comparing the numerical results to the exact results. This comparison is quantified with error and convergence rate.

## Results and model validation

In this section, we implemented our proposed scheme [12] to study the two-dimensional standing waves. For the numerical simulation, we take the computational domain as x∈ $\Omega_{p}=\left[0,2\right]\times \left[0,2\right],\;d\left(x\right)=d_{0}=0.5$ which is triangularly discretized. Discretization produces 681 nodal points with a total of 1280 triangular elements. Simulations are conducted using hard-wall boundary conditions with two kinds of initial conditions.

(16) $$\eta \left(x,0\right)=A\cos\left(k_{x}x\right),$$

(17) $$\eta \left(x,0\right)=A\cos\left(k_{x}x\right)\cos\left(k_{y}y\right),$$

with zero velocity potential

(18) ϕ(x,0)=0,

where A represents amplitude. The wave numbers corresponding to the sloshing motion in the x and ydirections are given by $k_{x}=\pi /L_{x},k_{y}=\pi /L_{y}$. Meanwhile, $L_{x}\;$and $L_{y}$ represent the length of the computational domain in the x or y direction. Here $L_{x}=L_{y}=2$. Furthermore, the analytical solution of standing waves in a closed basin is given by

(19) η(x,t)=η(x,0)cosωt,

(20) $$\phi \left(x,t\right)=-\frac{\text{Lg}}{\pi c}\cos\left(kx\right)\sin\omega t,$$

where $c=\sqrt{gd_{0}}\;$and ω is wave frequency given by the exact dispersion relation

(21) $$\frac{\omega^{2}}{k}=g\tanh kd,\;k=\sqrt{k_{x}^{2}+k_{y}^{2}}$$

Here simulation is linear and dispersive since $kd_{0}\gg \pi /10$ (see, Tarwidi et al. [32] for more discussion about dispersive waves).

**Fig. 2:**
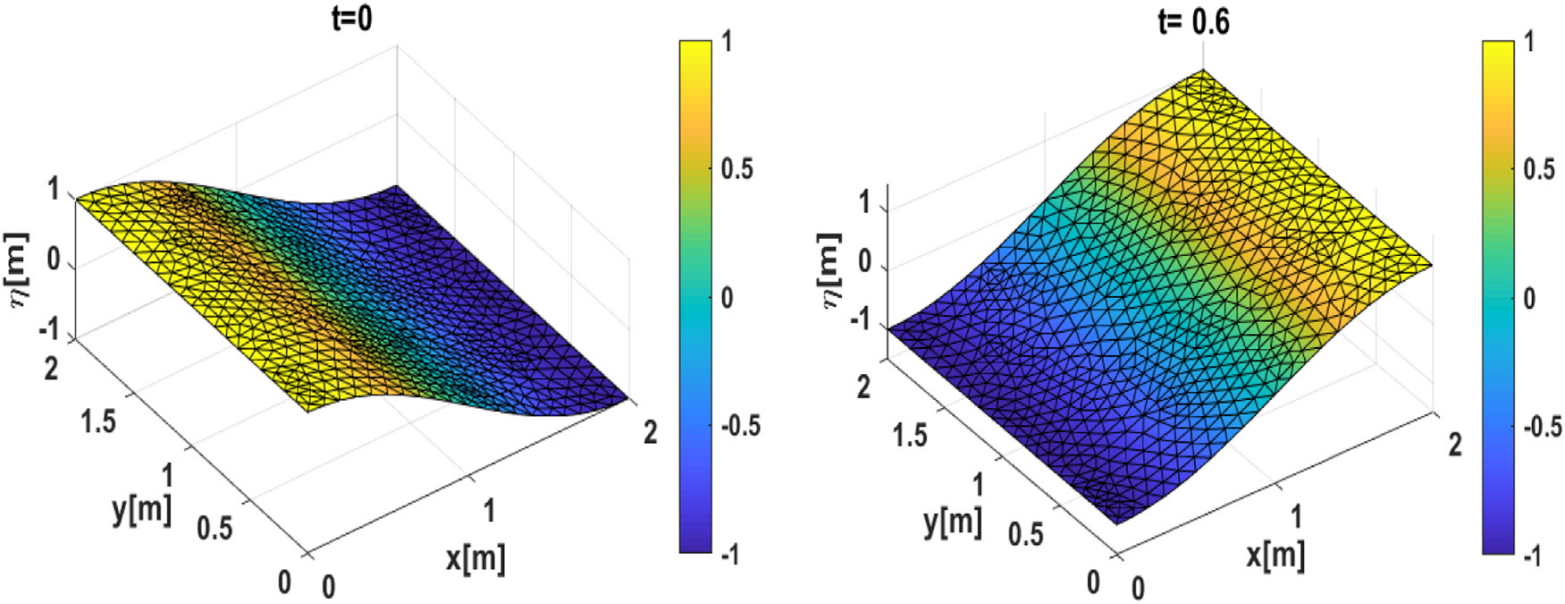
3D Plot the numerically free surface of the first simulation. (Left) Initial condition as given in [20] and [24], (Right) free surface η(x,t=0.6).

**Fig. 3:**
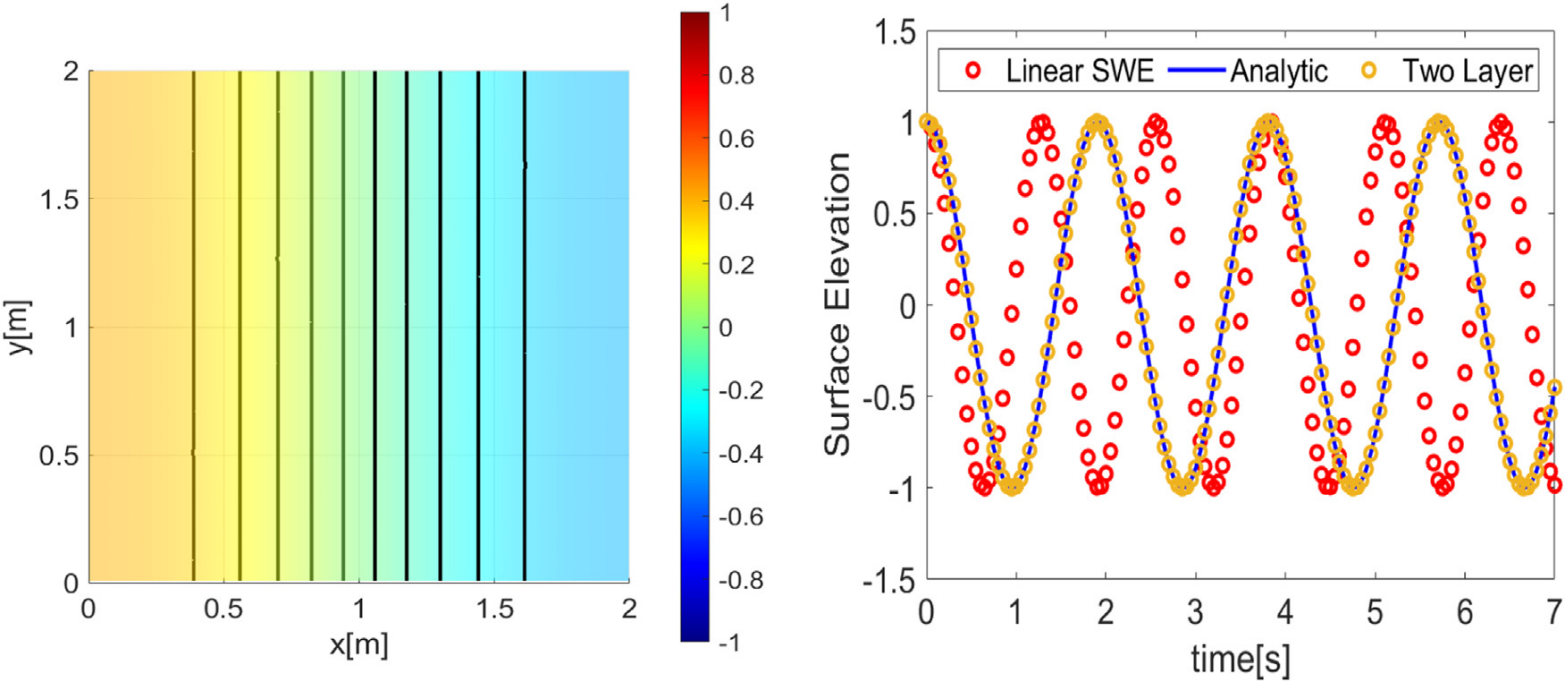
(Left) The contour of the numerical free surface plotted with the analytical solution (solid line) of the first simulation, (Right) time series of the free surface compared to the analytical and two-layer approaches at the position(x,y)=(0,0).

**Fig. 4:**
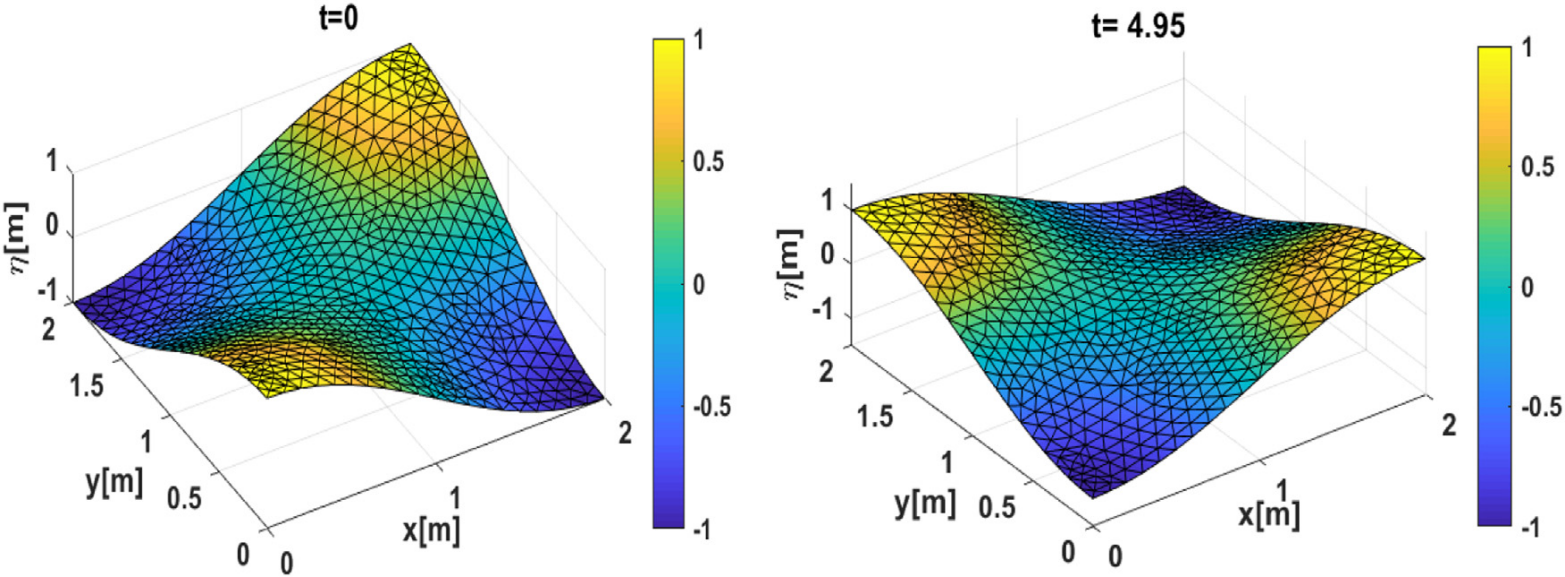
3D Plot the numerically free surface of the second simulation. (Left) Initial condition as given in [12] and [24], (Right) free surface η(x,t=4.95).

**Fig. 5:**
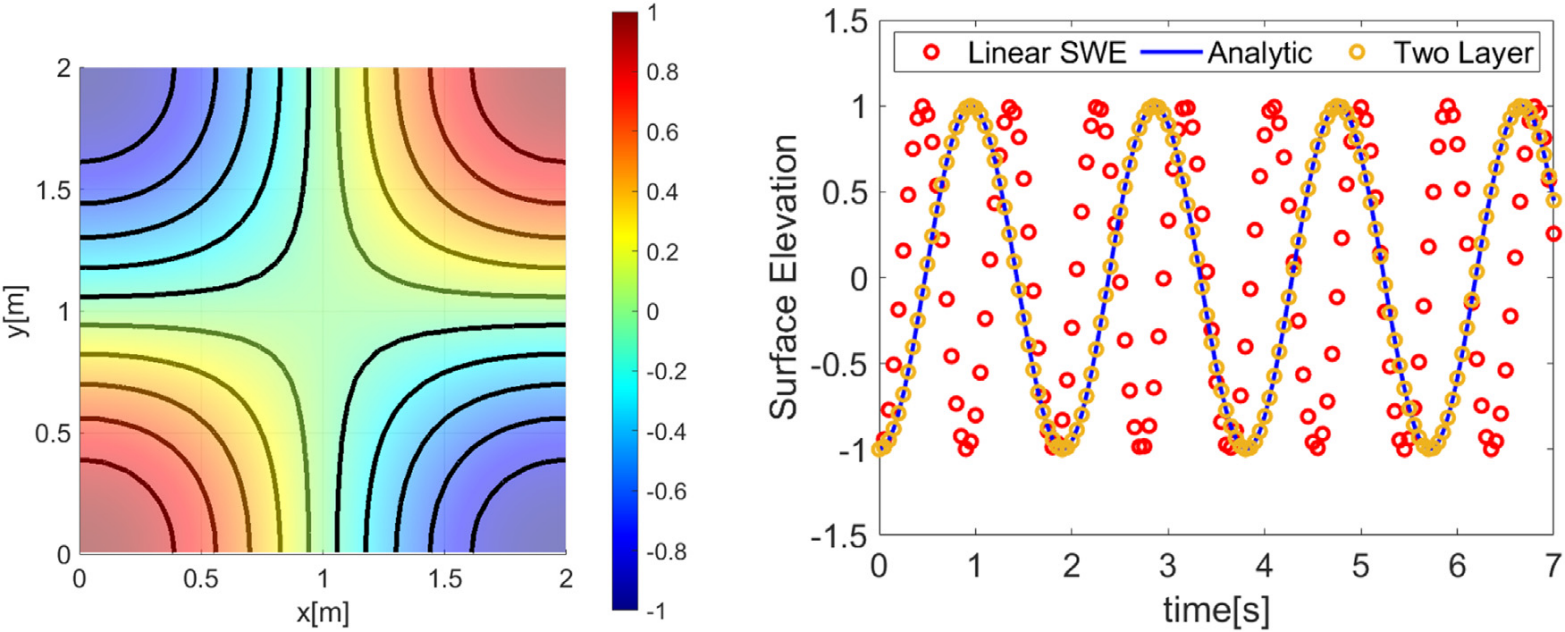
(Left) The contour of the numerical free surface plotted with the analytical solution (solid line) of the second simulation, (Right) time series of the free surface compared to the analytical and two-layer approaches at the position(x,y)=(0,−1).

Our first simulation results using the first initial condition; [20] and [24] are presented in Fig. 2, Fig. 3. Meanwhile, the second simulation using initial conditions; [12] and [24] is presented in Fig. 4, Fig. 5. The numerical free surface contour plots together with the analytical solutions in each case are given in Figs. 3 **(Left)** and **5 (Left)**, for the first and second simulation cases respectively. The color scale indicates the height of the free surface. The free surface oscillates without changing its shape and as we expected, the solutions stay symmetric around the lines x=1 for the first simulation and stay symmetric around the diagonals for the second simulation. The time series plot of the free surface at a specific location is given in Figs. 3 **(right)** and **5 (right)**. Note that, for the first simulation, the wave has a period $T_{num}=\;1.3$. So, the wave frequency is calculated as $\omega_{num}\;=\frac{1}{T_{num}}=\;\approx \;0.7692\;Hz$. For the second simulation, the wave has a period $T_{num}=\;0.9$. So, the wave frequency is calculated as $\omega_{num}\;=\frac{1}{T_{num}}=\;\approx \;1.1111\;Hz$.

Moreover, if we plot the exact frequency ω as given in [5] together with our numerical results and two-layer approach as in Tarwidi et al. [32], we can see that some discrepancies were found. It is because our numerical scheme is one layer, hydrostatic and linear, where the advection term is negligible. If we want to produce the exact result, then two layers with a hydrodynamic approach must be used. But overall, good agreement is found within our numerical results and exact solution, where the amplitude is preserved equally to one, which means our proposed scheme is free from numerical damping.

Furthermore, the numerical errors and convergence rate of our proposed scheme are summarized and presented in Table 1. We calculate the numerical error using the following formula:

$$E=\frac{1}{\max\left(N_{S},N_{V}\right)}\sum_{k=1}^{\max\left(N_{S},N_{V}\right)}\left|E_{k}^{num}-E_{k}^{exact}\right|$$

where $E_{k}$ denoted the corresponding value at k position. Meanwhile, the convergence rate C is given as

$$C=\frac{\log\left(E_{j}/E_{j-1}\right)}{\log\left(\Delta x_{j}/\Delta x_{j-1}\right)}$$

for the j,j−1 steps. If the number of partitions is increased, the error will be smaller by less than 1 %. From Table 1**,** it can be inferred that the proposed scheme has a convergence rate of the first order.

**Table 1:** Numerical error and convergence rate of the free surface and velocity potential.

$\times N_{S}$	$E_{\eta }$	$C_{\eta }$	$E_{\phi }$	$C_{\phi }$
11×11	0.1917245	–	0.1012450	–
21×21	0.0913225	1.147153	0.0812215	1.132153
101×101	0.0099012	1.040883	0.0090012	1.040883
201×201	0.0036651	1.025336	0.0012110	1.010016
1001×1001	0.0000951	0.996321	0.0000999	1.000032

### Dam-break planar 2D

**Fig. 6:**
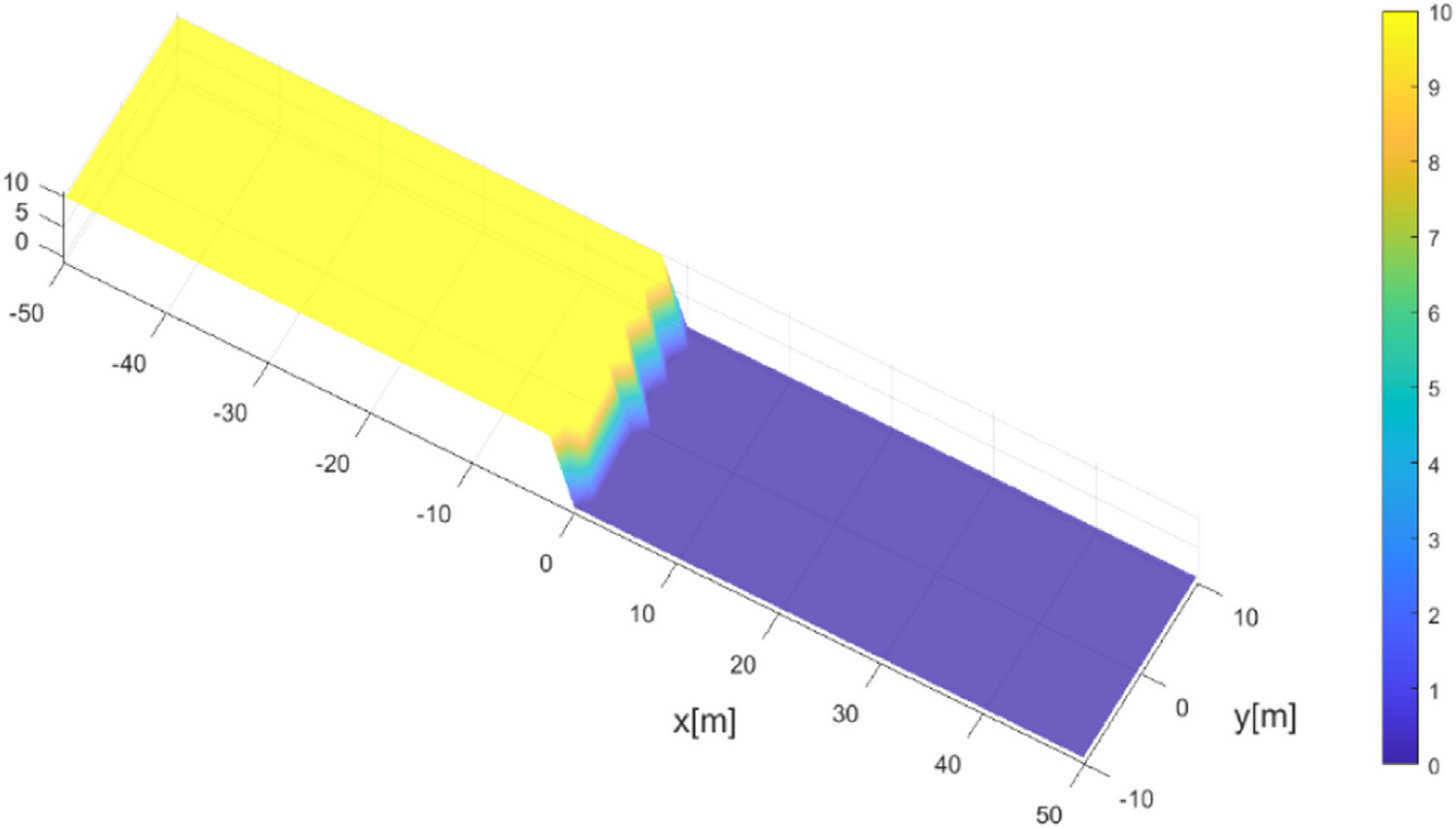
Initial condition for dam-break planar 2D.

Suppose we consider a flat topography without friction on a computational spatial domain given by $\Omega_{p}=\left(x,y\right)\in \left[-50,50\right]\times \;\left[-10,10\right]$. In this subsection, we aim to simulate the planar dam break with a wet dry area of 10 m for each side where the dam is located (0,y). The profile of the initial condition is plotted in Fig. 6 and set as follows.

$$h\left(x,t\right)=\left\{ \begin{array}{c} 10,\;x<0\\ h_{thin},\;x\geq 0 \end{array}\right.$$

ϕ(x,t)=0,

where $h_{thin}$ is a threshold value given beforehand. This procedure is a thin layer technique that represents a small layer of water in a dry area.

**Fig. 7:**
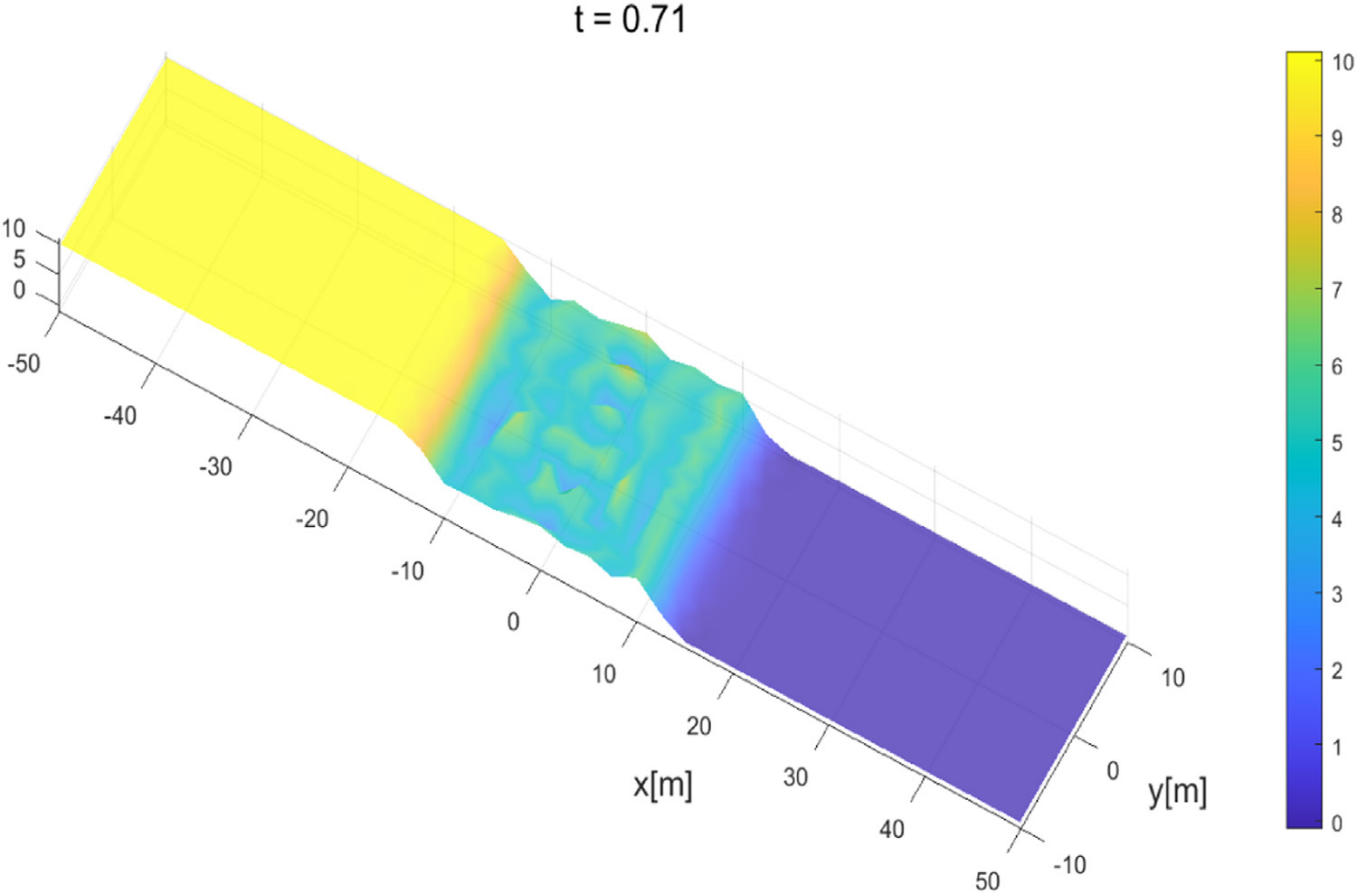
Water surface evolution for dam-break planar 2D at t=0.71.

**Fig. 8:**
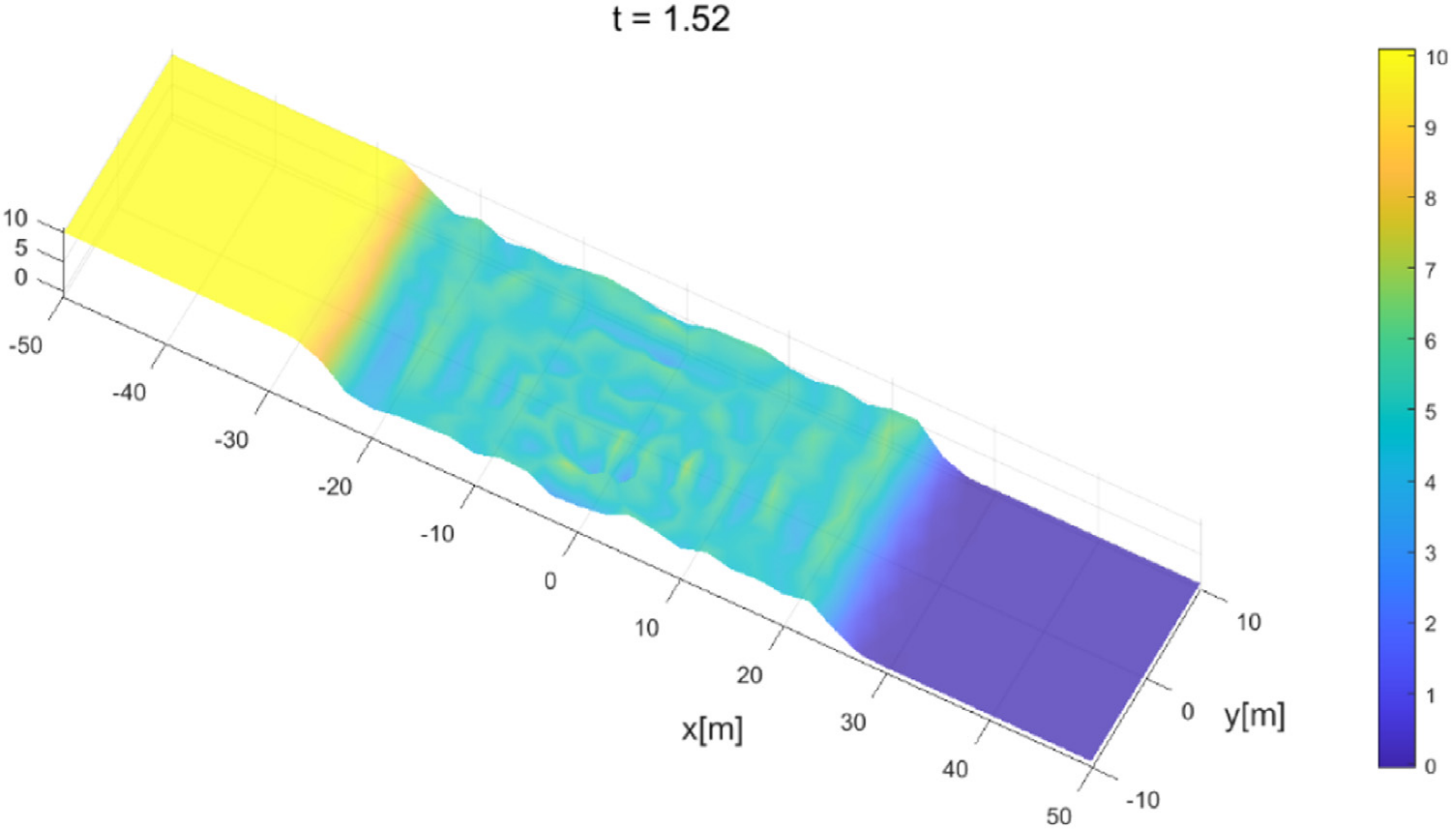
Water surface evolution for dam-break planar 2D at t=1.5.

After the dam collapsed, the profiles at a subsequent time are presented in Figs. 7 and 8. The water flow moves downstream due to the collapse of the dam wall. During its propagation, the water flows through a dry area that has been numerically treated with the wet-dry procedure. In general, the results obtained agree with the results obtained by Mungkasi and Roberts [29] using ANUGA and also agree with exact solutions, which is when at t=1.5, the water flow was 30 m away from the gate position.

### The effect of absorbing boundary

Suppose we consider the radiation boundary condition (absorbing), given as

(22) $$\phi_{t}\;=\;\pm \;c\;\nabla \;\phi \cdot \;n$$

where n is a normal vector. Then the weak form [11] must be modified. If we expand the integral in [11] using Gauss Theorem, then we have

(23) $$\int_{\Omega_{p}}\partial_{t}\eta Vd\Omega_{p}=-d_{0}\int_{\partial \Omega_{p}}\nabla \phi Vd\partial \Omega_{p}+d_{0}\int_{\Omega_{p}}\nabla \phi \nabla Vd\Omega_{p}.\;$$

In [11], the first term on the RHS of [2] becomes vanished because ∇ϕ on the boundary $\partial \Omega_{p}$ equals zero due to the hard-wall boundary. If we employ the RBC as given by [29], then the first term in the RHS of [2] cannot vanish and becomes

(24) $$\int_{\Omega_{p}}\partial_{t}\eta Vd\Omega_{p}=-d_{0}\left(\mp \int_{\partial \Omega_{p}}\frac{\partial_{t}\phi }{\sqrt{gd\left(x\right)}}V\partial \Omega_{p}\right)+d_{0}\int_{\Omega_{p}}\nabla \phi \nabla Vd\Omega_{p}.\;$$

So, again, we reformulated the weak form of SWE with RBC given as *determining* η∈E *and* ϕ∈P *such that*

(25) $$\int_{\Omega_{p}}\left(\partial_{t}\eta V-d_{0}\nabla \phi \nabla V\right)d\Omega_{p}-\left(\mp \sqrt{gd_{0}}\int_{\partial \Omega_{p}}V\eta d\partial \Omega_{p}\right)=0,\;\forall V\in E$$

(26) $$\int_{\Omega_{p}}\left(\partial_{t}\phi +g\eta \right)Wd\Omega_{p}=0,\;\forall W\in P$$

The previous expression [9] is obtained after considering [1]. Thus, the discrete form of the weak formulation of [9] and [17] is given by

(27) $$\left(\begin{array}{cc} M & 0\\ 0 & M_{\mathrm{nc}} \end{array}\right)\partial_{t}Y\left(t\right)=\left(\begin{array}{cc} \mathrm{BC} & S\\ -gM_{\mathrm{nc}} & 0 \end{array}\right)Y\left(t\right)$$

where the boundary matrix formulated as

$$\mathrm{BC}=\left[b_{kj}\right],\;b_{kj}=-\sqrt{gd_{0}}\int_{\partial \Omega_{p}}T_{k}\left(x\right)T_{j}\left(x\right)d\partial \Omega_{p}.$$

By using Crank Nicholson time integration to solve [28], we obtain

(28) $$\left(\begin{array}{cc} M-\mathrm{BC}\frac{\Delta t}{2} & -\frac{\Delta t}{2}S\\ -\frac{g\Delta t}{2}M_{\mathrm{nc}} & M_{\mathrm{nc}} \end{array}\right)Y\left(t\right)=\left(\begin{array}{cc} M+\mathrm{BC}\frac{\Delta t}{2} & \frac{\Delta t}{2}S\\ -\frac{g\Delta t}{2}M_{\mathrm{nc}} & M_{\mathrm{nc}} \end{array}\right)Y\left(t\right)$$

### Simulation of absorbing effect

In this section, our proposed scheme [10] will be used to simulate the water drop with an absorbing boundary [29] in four directions.

**Fig. 9:**
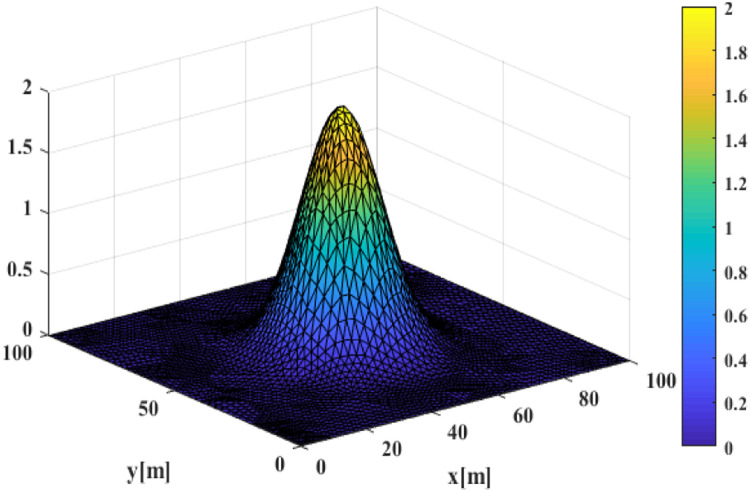
Initial Gaussian Profiles.

Simulations are conducted using $\Omega_{p}=\left[0,\;100\right]\times \;\left[0,\;100\right]$, $d_{0}=10$ and the triangulation process produces a total of $N_{S}=2731$ and $N_{V}=5296\;$. The initial value is fluid at rest with surface elevation given by Gaussian profiles.

$$\eta \left(x,t\right)=\;2\exp\left[\frac{{\left(x-x_{mid}\right)}^{2}+{\left(y-y_{mid}\right)}^{2}}{{\left(x_{mid}/4\right)}^{2}}\right]$$

as plotted in Fig. 9. This initial value is placed in the middle of the computational domain $\left(x_{mid},y_{mid}\right)\in \Omega_{p}$ with zero velocity potential. The waves will propagate throughout the computational domain and at the boundary, they will be absorbed in the four ends of the wall.

**Fig. 10:**
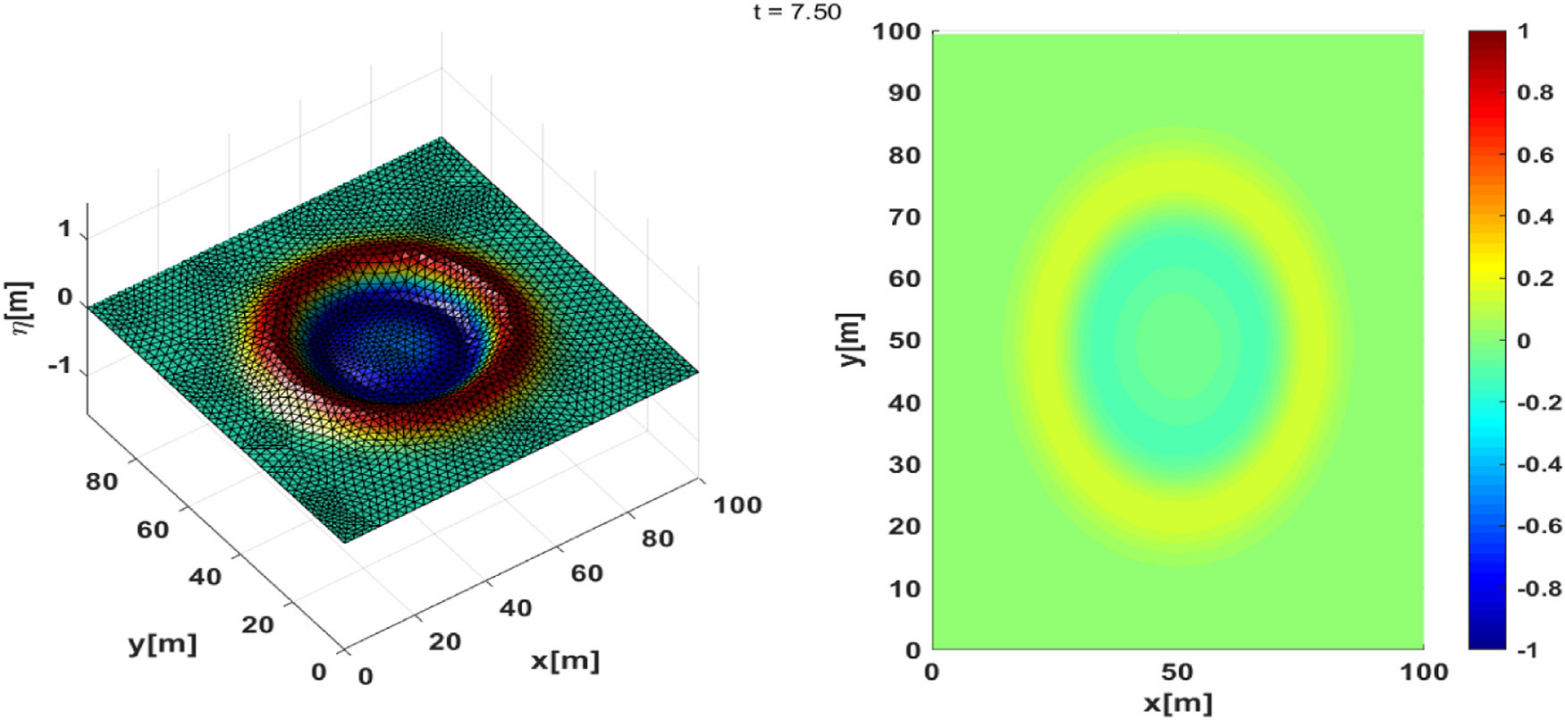
(Left) 3D Plot the numerically free surface at t=7.5, (Right) top view.

**Fig. 11:**
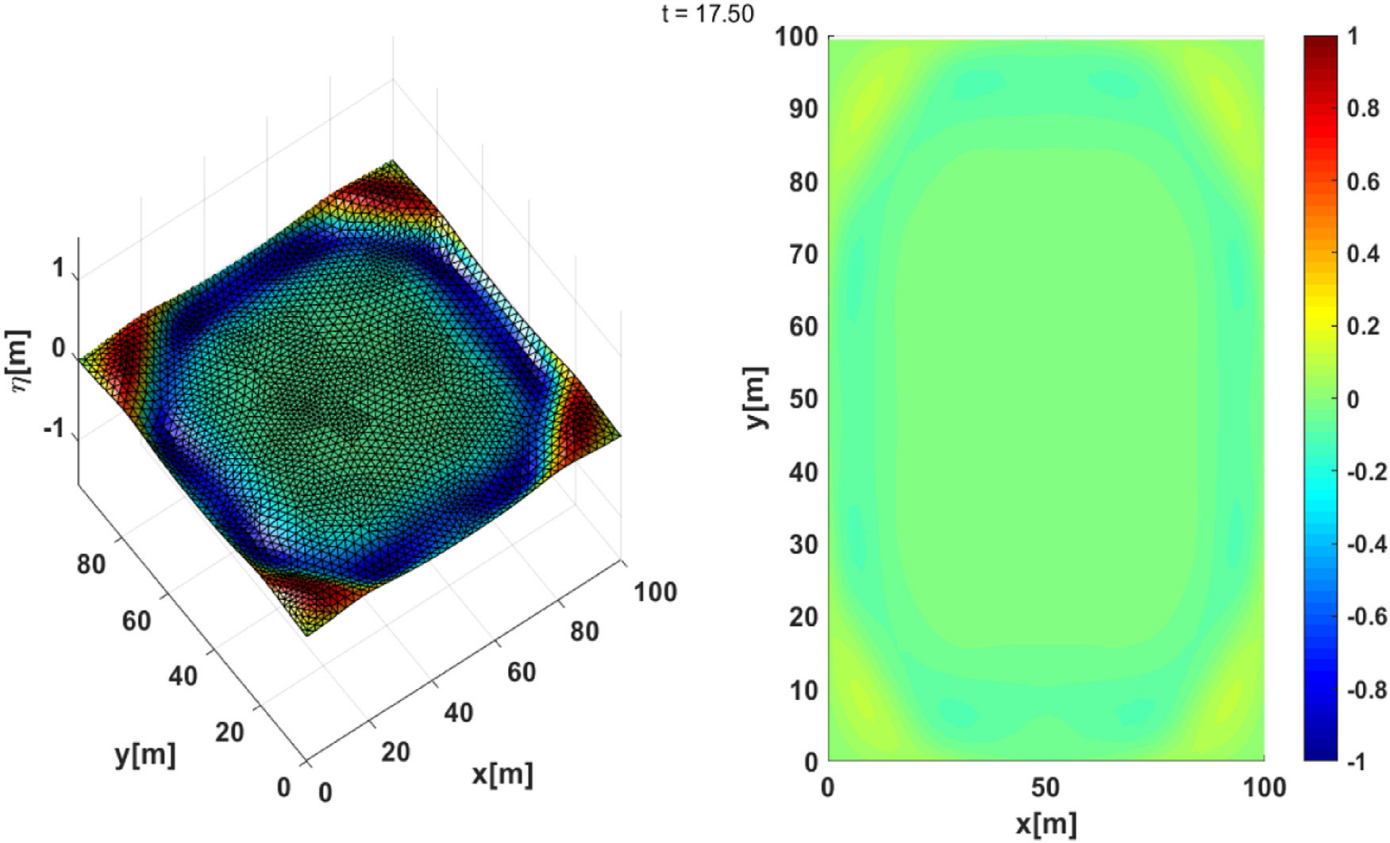
(Left) 3D Plot the numerically free surface at t=17.50, (Right) top view.

**Fig. 12:**
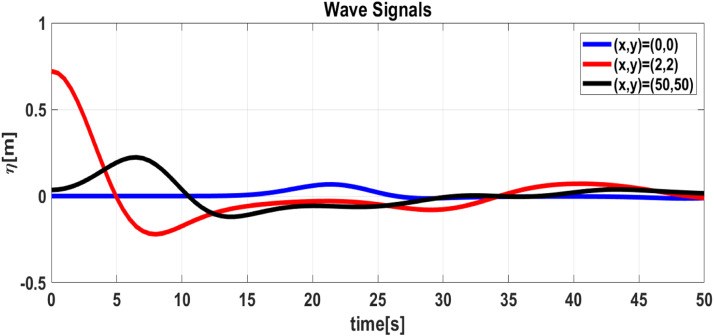
Time series of the free surface in three specific locations (x,y)=(0,0);(2,2);(50,50) during the absorbing simulation.

These results are presented in Figs. 10 and 11. We can see that the absorbing boundary conditions can absorb waves propagating towards the boundary, although there is still a slight reflection towards the computational domain, as shown in Fig. 11. We can see that the application of absorbing boundary conditions has been successfully implemented. The time series plot shows that in several places where the surface height is recorded, the reflected wave still emerges from the boundary, although it is quite small compared to the horizontal scale. Until the simulation is finished, the waves that propagate towards the boundary are well absorbed, as shown by the free surface time-series in Fig. 12**,** which vanishes towards zero.

## Conclusion

As an extension of our prior work, we have provided a novel approach to solving two-dimensional SWE using $P_{1}^{NC}-P_{1}$ finite element pairs. We have succeeded in solving the two-dimensional SWE equation in its primitive form, which is different from the common approach. In this case, the free surface is approximated by a conformal basis $P_{1}$, but the velocity potential is approximated by a non-conformal basis $P_{1}^{NC}$. As a result, the weak formulation is more flexible, and the discrete form is easy to construct. The resulting scheme is first-order ordinary differential system, which is easily solved by the Crank Nicholson time integration. Validation using analytical standing waves solution produced good accuracy with first-order accuracy. Although some discrepancies were found regarding frequency due to limitations of our one-layer, linear, hydrostatic model, we found that our numerical solution is free from damping errors. In addition, simulation of dam-break planar 2D cases shows good agreement with ANUGA software by Mungkasi et al. [29]. The successful implementation of the radiation boundary condition (RBC) demonstrates the flexibility of our scheme. The time series plot of surface height from the wave absorbing simulation reveals that the reflected wave still emerges from the boundary, however it is relatively small in comparison to the horizontal scale. Thus it can be neglected. All simulations provided in this research required no special treatment other than the precise implementation of the radiation boundary condition. All these findings have shown the robustness and capability of our scheme to predict accurate results for various problems. Our proposed $P_{1}^{NC}-P_{1}$ finite element pairs will be improved in future research on wave influx as well as the implementation in nonlinear SWE and the non-hydrostatic two-layer case.

## CRediT authorship contribution statement

**Putu Veri Swastika:** Conceptualization, Methodology, Validation, Visualization, Formal analysis, Writing – original draft. **Muhammad Fakhruddin:** Supervision, Writing – review & editing. **Sofihara Al Hazmy:** Conceptualization, Methodology. **Siti Fatimah:** Writing – review & editing. **Amaury de Souza:** Writing – review & editing.

## Declaration of Competing Interest

The authors declare that they have no known competing financial interests or personal relationships that could have appeared to influence the work reported in this paper.

## Data Availability

No data was used for the research described in the article.
